# A Case Report of Internet Gaming Disorder Treated With Bupropion and Cognitive Behavioral Therapy

**DOI:** 10.7759/cureus.63013

**Published:** 2024-06-24

**Authors:** Kasireddy Sravanthi, N. G. Nihal, N. N. Raju, Shailaja Mane

**Affiliations:** 1 Pediatrics, Dr. D. Y. Patil Medical College, Hospital & Research Centre, Dr. D. Y. Patil Vidyapeeth (Deemed to be University), Pune, IND; 2 Psychiatry, Gayatri Vidya Parishad Institute of Health Care and Medical Technology, Visakhapatnam, IND

**Keywords:** excessive gaming, cognitive-behavioral therapy (cbt), bupropion, adolescent addiction, internet gaming disorder (igd)

## Abstract

Gaming disorder is a growing concern, recognized by the World Health Organization and included in the Diagnostic and Statistical Manual of Mental Disorders, Fifth Edition (DSM-5) as internet gaming disorder (IGD) for further study. This case report describes a 13-year-old boy diagnosed with IGD according to the proposed DSM-5 criteria. The patient exhibited excessive gaming behavior leading to impaired academic performance and social interaction. Treatment included medication with bupropion and cognitive behavioral therapy (CBT) resulting in significant improvement in gaming habits and social functioning. This case highlights the effectiveness of a combined approach for managing IGD and emphasizes the need for further research to optimize treatment strategies.

## Introduction

Video games have become a major source of entertainment for young people around the world. However, while video games offer enjoyment and stimulation for many, a subset of individuals experiences a concerning shift towards addictive and potentially debilitating behaviors [1].

Gaming disorder has recently been proposed for inclusion in the 11th Revision of the International Classification of Diseases (ICD-11) [2]. Additionally, the 5th edition of the Diagnostic and Statistical Manual of Mental Disorders (DSM-5) has already included internet gaming disorder (IGD) in the section for conditions requiring further study [3]. Research suggests a global prevalence of IGD as high as 3.1% [4], with a wider range observed in India (3.6% to 23.0%) among adolescents and young people [5].

Research consistently indicates that males and individuals under 19 are at higher risk for IGD [6-8]. Therefore, in this case report, we demonstrate the effectiveness of a combined approach for managing IGD and emphasize the need for further research to optimize treatment strategies.

## Case presentation

A 13-year-old boy studying in class 8 is the only child in a nuclear family with both parents working. His temperament is described as easygoing.

During early childhood, the boy's parents often gave him a cell phone to keep him engaged while they were at work. He frequently watched videos or played games on the device. Over the past year, his parents observed a significant increase in his online gaming duration, escalating from one to two hours to six to eight hours daily. This excessive gaming interfered with his school assignments, leading to a noticeable decline in his scholastic performance, which became the main concern for his parents.

When attempts were made to curtail his gaming time, the boy would become stubborn and refused to attend school. He exhibited irritability and argumentativeness when limits were imposed on his gaming activities. He began preferring time spent on gaming over other activities, leading to a lack of self-confidence and an inferiority complex when interacting with peers and teachers. His social interactions with relatives also deteriorated, and he appeared progressively socially detached, eventually abandoning school to spend more time gaming. There was no past or family history of psychiatric illness.

The boy was assessed using the Internet Gaming Disorder Scale-Short Form (IGDS9-SF) and obtained a score of 31. A general physical examination revealed that his overall condition, sensory, and vital signs were within normal limits. During the interview, the boy revealed low self-esteem but reported preserved sleep and appetite, with no delusions. He mentioned that playing online games provided him with a sense of connection.

Differential diagnoses of depression, anxiety, and attention deficit hyperactivity disorder (ADHD) were ruled out. A provisional diagnosis of IGD was made according to the DSM-5 criteria. The boy was treated with bupropion 150 mg/day and underwent cognitive behavioral therapy (CBT) sessions. The management plan included a gradual reduction of gaming time, encouragement to explore other areas of interest and indoor activities, and providing a safe environment for the boy to express his feelings. Additionally, psychoeducation for the parents about IGD and strategies to improve their interaction with their child was provided.

Weekly sessions were conducted for the first month, followed by bi-weekly sessions thereafter. After eight weeks, the boy showed progressive improvement. He started enjoying his usual interests with family members and actively sought social interactions, including planning lunches with adolescents he met at the hospital. His gaming time was reduced to approximately one to two hours per day.

## Discussion

**Figure 1:**
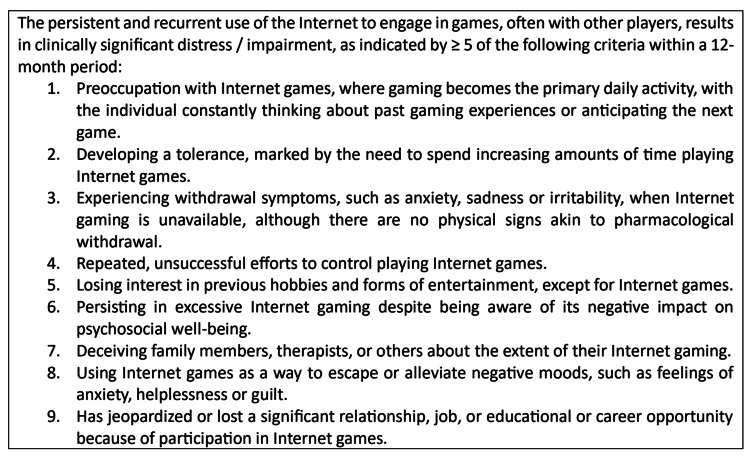
Proposed DSM-5 criteria for internet gaming disorder This figure shows the proposed criteria by DSM-5 of the American Psychiatric Association [3]. DSM-5: Diagnostic and Statistical Manual of Mental Disorders, Fifth Edition

Figure 1 lists the nine proposed criteria for IGD by the American Psychiatric Association. The patient profiled in this case study satisfied every diagnostic requirement outlined in this proposed definition of IGD. Over time, he played video games more frequently, going from one to two hours per day to six to eight hours. His scholastic performance declined as a result of his video game addiction. Furthermore, resulted in impaired social interaction with peers and relatives, but he still continued to use it. He becomes irritable and argumentative when attempts are made to limit his gaming time. He gave up school so he could continue gaming.

Several studies have linked excessive gaming with negative consequences including sleep problems, social isolation, relationship strain, job loss, poor diet, and physical inactivity. Additionally, excessive gaming may contribute to feelings of grief, social detachment, intellectual decline, and body image dissatisfaction [9,10].

The IGDS9-SF is a brief psychometric tool consisting of nine items that align with the nine core IGD criteria outlined in the DSM-5 used for the assessment of IGD. Research indicates that these items collectively form a single-factor structure and provide reliable and valid measures for assessing IGD. The IGDS9-SF has demonstrated solid construct and criterion validity and with good internal consistency, evidenced by Cronbach's α of 0.88 [11]. It gauges the severity and negative impacts of IGD by examining both online and offline gaming activities over the past year. A 5-point Likert scale is used, where 1 means "Never," 2 means "Rarely," 3 means "Sometimes," 4 means "Often," and 5 means "Very Often." The overall score, which ranges from 9 to 45, is derived by summing the scores of all items, with higher scores indicating more severe IGD [12].

While treatment research for IGD is ongoing, there are promising approaches with preliminary support. These include CBT, medications like bupropion and escitalopram, transcranial direct current stimulation (tDCS), mindfulness-based therapies, and family therapy. Current therapeutic interventions for IGD remain in their infancy, and the identification of empirically validated treatment strategies necessitates further investigation [13].

CBT has been proposed as an effective treatment for IGD. It aids individuals in addressing maladaptive thoughts and behaviors, as well as social and behavioral issues, and motivates them to make positive changes and adopt alternative behaviors. The core principle of CBT is that behaviors stem from people's perceptions, thoughts, and beliefs. Through CBT, individuals learn to identify harmful cognitive patterns, replace them with healthier ones, and modify their fundamental beliefs. When an individual's beliefs about something change, their behavior will also change accordingly [14].

Bupropion was the most commonly used medication in treating IGD. Considering that IGD is viewed as a behavioral addiction and often co-occurs with depression, anxiety, and ADHD, this is not unexpected. Bupropion has proven effective in treating smoking cessation, depressive disorders, anxiety symptoms, and ADHD. These findings may shed light on the neurobiological mechanisms by which bupropion acts in IGD, particularly given the shared pathophysiology involving dysregulated dopaminergic signaling in these conditions and bupropion's role in regulating dopamine release [15].

## Conclusions

Over time, the prevalence of IGD, a potentially new behavioral addiction, is expected to rise. This case underscores the importance of early recognition and comprehensive management of IGD. Combined pharmacological and behavioral interventions, particularly CBT, appear effective in addressing the multifaceted challenges associated with IGD. Further research is recommended to standardize diagnostic criteria and optimize treatment strategies for this emerging disorder.

## Figures and Tables

**Figure 2:**
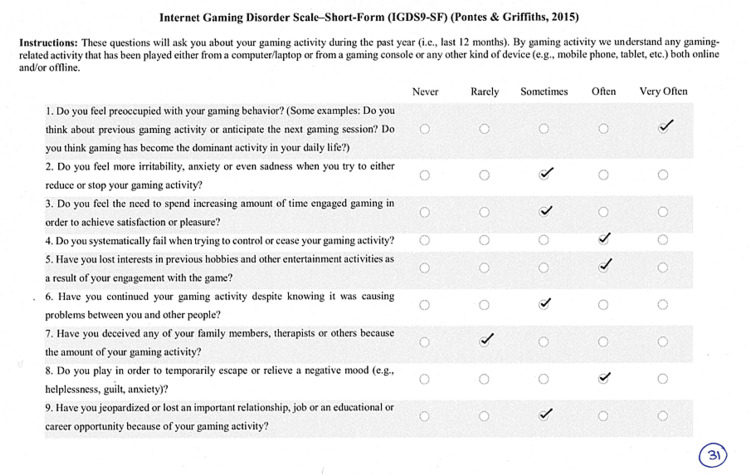
Scoring of the Internet Gaming Disorder Scale-Short Form (IGDS9-SF) for a 13-year-old boy
